# Optimization of harvest and extraction factors by full factorial design for the improved yield of C-glucosyl xanthone mangiferin from *Swertia chirata*

**DOI:** 10.1038/s41598-021-95663-7

**Published:** 2021-08-11

**Authors:** Prabhjot Kaur, R. C. Gupta, Abhijit Dey, Tabarak Malik, Devendra Kumar Pandey

**Affiliations:** 1 Department of Biotechnology, School of Bioengineering and Biosciences, Lovely Professional University, Phagwara, Punjab 144411 India; 2 Department of Botany, Punjabi University, Patiala, Punjab 147002 India; 3 Department of Life Sciences, Presidency University, Kolkata, 700073 India; 4 Department of Biochemistry, College of Medicine and Health Sciences, University of Gondar, Gonder, Ethiopia

**Keywords:** Drug discovery, Plant sciences

## Abstract

*Swertia chirata* Buch.-Ham. ex C.B. Clarke is an important medicinal plant used in various herbal formulations as it shows significant biological activities such as hepatoprotective, hypoglycemic, anti-inflammatory, antimalarial, antioxidant and anti-parkinson. C-glucosyl xanthone glycoside (mangiferin) is known as bio-marker compound of genus *Swertia* L. Development of efficient extraction methods of C-glucosyl xanthone mangiferin from *Swertia chirata* was attempted by optimizing the pre-harvest, post-harvest and extraction techniques by full factorial design. Firstly, a full factorial design was implemented to evaluate the single and interactive effects of pre-harvest (growth stage and plant part), post-harvest (drying condition and storage periods) followed by selection of best extraction technique such as heat reflux extraction (HRE), microwave assisted extraction (MAE) and ultrasound assistant extraction (UAE) at different solvent types on mangiferin yield. HPTLC and HPLC techniques were used for the determination of mangiferin content in extracts generated from different plant samples. In addition, anti-oxidant and anti-diabetic properties were determined by using DPPH assay and percentage inhibition of α‑amylase enzyme. Substantial variation of mangiferin yield, ranged from 1.46 to 4.86% was observed, depending on the growth stage, plant part, drying condition, storage periods and extraction method. Results showed that drying of the leaves of *Swertia chirata* in the shade harvested at budding stage and stored for not more than 1 month was recommended for obtaining a higher mangiferin yield. Among different extraction techniques, MAE and UAE in 50% aqueous ethanol solvent were found to be efficient and cost-effective with better yield of mangiferin (4.82% and 4.86%, respectively) as compared to HRE (4.14%). Highest DPPH activity and percentage inhibition of α‑amylase was observed in the aqueous ethanol extract of *S. chirata* leaves harvested at bud-stage of plant followed by flowering stage. The study shows that optimization of various factors by full factorial design was found to be an effective procedure to improve mangiferin yield from *Swertia chirata* and can be used for extraction of mangiferin.

## Introduction

*Swertia chirata* Buch.-Ham. ex C.B. Clarke (Gentianaceae) is an important medicinal herb found in the high altitude region of tropical Asia, America, Africa and Europe within an altitude of 1200–3600 m. Till date, around 170 species of this important herb are discovered. In India, it is distributed mainly in North Eastern Himalayas (about 13–14 species), North Western Himalayas (around 16 species) and Western Ghats. Traditional system of medicine such as Ayurveda, Sidha and Unani, as well as modern research emphasized the potential of *S. chirata* as an important medicinal herb^1^. This herb is a major source of many bioactive phytochemicals such as mangiferin, amarogentin, swertiamarin, oleanolic acid and ursolic acid^2–6^. *S. chirata* is used as a principal component in several herbal formulations such as Mahasudarshan Churna (Dabur India Ltd), Diabecon (Himalayan Pvt. Ltd), Ayush-64 (NRDC), Melicon V-Ointment (Cadila Pharmaceutical Ltd), Menstryl Syrup (Dabur India Ltd)^7,8^. *Swertia* has been proven as an industrially important herbal medicine as its export marketing is growing by 10% annually^9^.

Nowadays, more than hundred xanthone compounds have been isolated from *Swertia* genus. Mangiferin, a C-glucosyl xanthone, is considered as one of the most significant medicinal xanthonoid, which can be used as an important phytochemical marker to screen the elite population of *Swertia*^3,4,10^. Mangiferin compound has been reported to exhibit many important pharmacological activities such as hepatoprotective^10^, anti-oxidant^11^, anti-diabetic^12^, anti-parkinson^13^, chemopreventive^14^, hypoglycemic^15^, cardiotonic and diuretics properties^16^. Garrido et al.^17^ have reported that mangiferin compound also helps to improve oxidative stress during neurodegenerative disorders.

The health benefits of herbal remedies are immense, but quality related problems seem to be masking the health benefits, which can be overcome by proper standardization methods of herbal materials. According to the guidelines developed by the United States food and drug administration (USFDA) and the European agency for the standardization of medicinal products (EMEA), certification of herbal products must be authenticated by various analytical procedures. The superiority of herbal medicines has been affected by influencing factors such as harvesting periods, proper sample drying, storage periods and an effective extraction methodology^18^. Thus, the collection at the optimum growth stage of the plant and harvesting month is extremely foremost phase before the sample preparation of herbal medicines. In addition, drying of plant samples by standardized method is very significant in industrial processing as chemical constituents degrade with microbial attack. Thus, desired bio-active compounds should be extracted within the proper time period to get the high content as the shelf-life of plant samples varies.

Mixtures of several multifarious bio-active compounds are present in plant extracts which may affect the sample preparation. Selection of efficient extraction technique is a major concern for the recovery and isolation of desired bio-active compounds from a mixture of phyto-constituents. In *Swertia chirata,* mangiferin has been extracted by various conventional techniques such as heat reflux extraction (HRE), maceration, shaking, percolation and Soxhlet^3,8,19,20^. The classical methods of extraction viz. Soxhlet and solvent extraction require relatively large quantity of solvent and are time consuming, causing a negative effect on activity of compound. On the other hand, modern green technologies are based on the miniaturization principles which employ physical action on the plant material on improving the extraction efficiency of bioactive compounds. Among these techniques, the most notable is ultrasound assisted extraction (UAE) and microwave assisted extraction (MAE) used to extract many bioactive compounds. UAE and MAE are performed for solvent extraction using ultrasound and microwave, respectively. These extraction methods are economical as well as eco-friendly and support the objective of green technology by reducing the extraction time and organic solvents^20^. UAE has been used to extract bioactive compounds from various plant materials such as *Curcuma amada*^21^ and *Mangifera indica*^22^. MAE has been used to extract bioactive compounds from *Mangifera indica*^23^ and many other plants.

Moreover, selection of solvent is an extremely important step for cost effective extraction of bioactive compounds from plants^24^. The polar fractions are extracted with aqueous methanol, which enables high extraction value^25^. Commonly used solvent for the extraction of polar compound, mangiferin in *Swertia chirata* is methanol^14^ whereas ethanol was used by only few researchers^19^. The full factorial design is a statistical tool which allows evaluating the significance of the effects and their interactions of a process. The classical optimization studies use the one-factor-at-a-time approach in which only one factor is variable at a time while the other parameter kept constant, which is time consuming and costly. Recently, a number of statistical designs with full factorial design have been employed for optimizing the yield of phytochemicals from plants^26^.

Presence of mangiferin has already been reported earlier in *Swertia chirata*^3,4^ but these studies did not elaborate the presence of mangiferin on the basis of organ, seasonal variation, drying conditions, solvent type and extraction techniques. Firstly, in the present work, a full factorial design 3 × 5 × 2 was carried out to assess mangiferin yield from growth stages (rosette, vegetative, bud, flower and fruiting), drying conditions (shade, sun and oven) and storage periods (first and sixth month). Secondly 3 × 5 full factorial design to optimize the extraction method and solvent types on best yield of mangiferin yield was estimated. As far as we know, no optimization studies on preharvest, postharvest conditions and extraction techniques were conducted on *Swertia chirata.*

## Methods

### Chemicals

Solvents and chemicals used for extraction were of analytical grade (Sigma, Aldrich), and those used for HPTLC and HPLC analysis were of HPLC grade. Standard mangiferin compound was obtained from Sigma Aldrich (USA).

### Plant material

*Swertia chirata* were purchased for research approach only from a local nursery propagating medicinal plants from the Chakrata region (Uttarakhand, India) (30.7016° N, 77.8696° E; altitude 2118 m) in different growth stages (rosette, vegetative, budding, flowering and fruiting stage) during the months of June, July, August, September and November in the year 2016. The plant was authenticated by author, Dr. D.K. Pandey on the basis of morphological characters and were identified by comparing with herbarium specimens in Herbaria of Botany Department, Punjabi University, Patiala and FRI, Dehradun. The Voucher specimens No. 11112016 were prepared and are present in the Department of Bioengineering and Biosciences of Lovely Professional University, Phagwara, Punjab, India. The leaf, stem and root parts were separated and thoroughly washed with tap water, cut into small pieces, shade dried and powdered separately in an electric grinder. High performance thin layer chromatography (HPTLC) method was to screen out the potent plant part of *S. chirata* containing mangiferin.

To study the effect of drying, three drying conditions i.e. sun drying, shade-drying and oven-drying were investigated. 50 g of fresh sample was spread over 1 m^2^ of the white sheet and kept under varying drying condition i.e. Hot air oven-drying (24 h at 45 °C), sun-drying (5 days at 35–45 °C) and shade drying (5 days at 30 °C). To study the effect of storage periods, dried samples of *S. chirata* were kept in polypropylene containers in dark conditions at room temperature for 1 and 6 months, respectively (Table 1).

**Table 1:** Effects of drying method and time of harvest (season and growth stage) on mangiferin yield from different storage periods of *Swertia chirata* L.

S. no.	Growth stage	Drying methods					
		Shade drying		Sun drying		Oven drying	
		Storage periods					
		First month	Sixth month	First month	Sixth month	First month	Sixth month
1	Juvenile	2.29 ± 0.07^d^	2.12 ± 0.02^d^	2.17 ± 0.07^d^	2.05 ± 0.01^d^	2.27 ± 0.03^d^	2.05 ± 0.03^d^
2	Vegetative	3.39 ± 0.07^c^	3.11 ± 0.01^c^	3.36 ± 0.02^c^	3.04 ± 0.01^c^	3.38 ± 0.03^c^	3.02 ± 0.14^c^
3	**Bud stage**	**4.73 ± 0.04**^**a**^	**4.34 ± 0.02**^**a**^	**4.66 ± 0.03**^**a**^	**4.25 ± 0.02**^**a**^	**4.72 ± 0.05**^**a**^	**4.31 ± 0.03**^**a**^
4	Flower stage	4.32 ± 0.02^b^	4.08 ± 0.02^b^	4.23 ± 0.14^b^	4.05 ± 0.03^b^	4.23 ± 0.09^b^	4.08 ± 0.06^b^
5	Fruit stage	3.45 ± 0.01^c^	3.11 ± 0.01^c^	3.42 ± 0.02^c^	3.05 ± 0.01^c^	3.46 ± 0.02^c^	3.11 ± 0.14^c^

Bold value indicate best results.

All analyses are the mean of triplicate measurements ± standard deviation. The results were analyzed by one-way ANOVA followed by Duncan’s Multiple Range Test (SPSS 16 was used for DMRT and Minitab 18 was used for full factorial design). Values with different superscript alphabet (a–d) within the same column are significantly different at *p* < 0.05.

### Preliminary experiments for screening of solvent types and efficacy of extraction techniques

For better solubility and extraction of mangiferin compound, suitable solvents such as chloroform, ethyl acetate, methanol, aqueous methanol (25–75% v/v), ethanol, aqueous ethanol (25–75% v/v) and water was screened with heat reflux extraction method. Aliquots (1 g) powdered leaves were heated at 75 °C temperature with 50 mL of different solvents for 1 h, according to Chavan et al.^27^, with slight modifications. All three extraction methods viz. HRE (75 °C for 1 h), MAE (450 W for 2 min.) and UAE (200 W, 40 KH, 40 °C) used in the present work are summarized in Table 2.

**Table 2:** Mangiferin content with respect of extraction technique, extraction time, and solvent types from leaves of *Swertia chirata.*

S. no.	Extraction methods	Solvent type	Time (min)	Mangiferin content (% dry wt.)
1	HRE	100% ethanol	60	1.46 ± 0.07
2	HRE	75% ethanol	60	1.77 ± 0.05
3	HRE	50% ethanol	60	4.14 ± 0.15
4	HRE	25% ethanol	60	3.13 ± 0.09
5	HRE	Aqueous	60	2.75 ± 0.09
6	UAE	100% ethanol	30	2.22 ± 0.09
7	UAE	75% ethanol	30	4.62 ± 0.17
**8**	**UAE**	**50% ethanol**	**30**	**4.86 ± 0.19**
9	UAE	25% ethanol	30	3.77 ± 0.12
10	UAE	Aqueous	30	3.21 ± 0.11
11	MAE	100% ethanol	**2**	2.14 ± 0.09
12	MAE	75% ethanol	**2**	4.52 ± 0.07
13	MAE	50% ethanol	**2**	4.82 ± 0.19
14	MAE	25% ethanol	**2**	3.77 ± 0.21
15	MAE	Aqueous	**2**	3.13 ± 0.11

Bold value indicate best results.

*wt*. weight.

All the extracts were filtered through Whatman No.1 filter paper, centrifuged for 4 min at 4 °C and supernatant was concentrated in Rota evaporator (Buchi model R-205). The residue (10 mg) was re-dissolved in 10 mL methanol and kept at 4 °C for further HPTLC and HPLC analysis. The stock solution of mangiferin reference compound was prepared in methanol solvent (10 mg per 100 mL) and then stock solution of the standard was stored at 4 °C.

### Experimental design and statistical analysis

Mangiferin extraction experiments for various samples of *Swertia chirata* were conducted in two steps, firstly a triplicate completely randomized 3 × 5 × 2 full factorial design arrangement of preharvest condition evaluating (rosette, vegetative, bud, flower, fruit stage) at three drying method (shade, sun and oven) and two storage periods (first and sixth month) a total of 30 sets were created and implemented in a random order. Secondly, 3 × 5 factorial design of extraction techniques evaluating three extraction methods (HRE, MAE and UAE) at five different levels of aqueous ethanol (0, 25%, 50%, 75%, 100%) in 15 extraction sets were randomly generated.

The mangiferin yield data were examined by analysis of variance (ANOVA) using the general linear model (GLM) approach of the MINITAB 18.0 software program (Minitab Inc, State College, PA, USA). The single and interactive effects of extraction factors on mangiferin yield were determined to examine the significance of differences at *p* < 0.05. The mean mangiferin yield was compared by Duncan’s multiple range tests. Graphics (main effect plots and interaction plots) were created with Minitab 16 software.

### Isolation of compound (flash chromatography, IR and NMR)

Mangiferin isolation was performed on a Biotage One Flash Chromatography system (Sweden), with UV detection and an automatic collector. For isolation of bio-active compound, the crude ethanolic extract (1 mg mL^−1^) prepared from the leaves of *Swertia chirata* by MAE was used. The Flash Chromatography conditions were as follows: 40 g flash column packed with 60–120 mesh sized silica gel; elution system: ethyl acetate/methanol gradient; wavelength: 254 and 280 nm and flow rate: 20 drop min^−1^. Before binding experiment, ethyl acetate was used to equilibrate the column former to isolation. Mangiferin was isolated in 40 min gradient program of solvent A (ethyl acetate) and solvent B (methanol). Out of seven fractions, mangiferin as the major peak was separated as first fraction (FA) after residue at wavelength 254 nm.

Structure of mangiferin compound in fractions was elucidated based on their spectral data (IR and ^13^C NMR). Purity of mangiferin compound was confirmed by comparison with reference compound (procured from Sigma Aldrich, USA) using HPTLC (high performance thin-layer chromatography) method.

### Quantification of marker compound by HPTLC and HPLC

#### HPTLC analysis

The HPTLC system was CAMAG (Muttenz, Switzerland) having Linomat-5 automatic sample applicator furnished with a 100 μL Hamilton syringe (fixed 100 nL s^−1^ delivery rate). For analysis, a twin-trough glass tank (CAMAG) and UV cabinet was used. Chromatography was performed on stationary phase composed of 20 cm × 10 cm pre-coated silica gel 60 F_254_ HPTLC plates (with 0.25 mm thickness). Samples were administered to the plates as 5 mm wide bands with Hamilton syringe. 3 µL plant samples were loaded on chromatographic plate. The pre-coated silica gel 60 F_254_ TLC aluminium plates was developed in 20 cm × 10 cm twin trough glass chamber saturated (20 min pre-saturation) with mobile phase ethyl acetate-glacial acetic acid-formic acid-water 100:11:11:26 (v/v/v/v) up to distance 7.5 cm at constant temperature (25 °C ± 2 °C) and relative humidity (40 ± 2%). The developed plate was dried by hot air to evaporate the solvents from the plate; bands were visualized and photographed with CAMAG visualizer under UV_254_ light. Scanning was completed with CAMAG TLC scanner-3 provided with CATS software (version: 1.4.4.6337) at λ = 254 nm. The determination of mangiferin in *Swertia chirata* samples were carried out by using high-performance thin layer chromatography (HPTLC) by following the method developed by Pandey et al.^28^, with slight modifications.

#### HPLC analysis

Analysis was performed with HPLC–PDA detector System (Waters) equipped with an auto sampler, a dual low-pressure gradient system, C-18 column. HPLC conditions were optimized with isocratic elution; 9 min time run and 1 mL min^−1^ flow rate. Working solutions of the samples were prepared at 100 ppm concentrations, and injection volume was set at 10 µL. Before injection, column was equilibrated for 10 min.

Chromatographic separation was carried out using Sunfire C-18 column (4.6 mm × 250 mm; 5 µm particle size). The mobile phase was composed of acetonitrile (A) and 0.1% formic acid in water (B) using the following gradient program: 20–46% A (0–1.0 min); 46–46% A (1.0–2.5 min); 46–20% A (2.5–4.0 min) and 20–20% A (4.0–9.0 min). UV–vis spectra were observed within the range of 200–400 nm.

### Preparation of calibration curve of mangiferin and validation of the developed method

For preparation of calibration curves of mangiferin, different concentrations of working standard solution [2 μL (200 ng), 4 μL (400 ng), 6 μL (600 ng), 8 μl (800 ng)] were applied to obtain linearity range of 200–800 ng spot^−1^.

Method validation was performed on the parameters such as linearity, limit of sensitivity, specificity, precision, accuracy, recovery and robustness.

### Anti-oxidant activity assay

#### 1,1-Diphenyl-2-picrylhydrazyl (DPPH) assay

The DPPH assay was used in order to evaluate the free radical scavenging activity of leaf parts of *S. chirata* harvested at bud- and flower-stages of plant. Extracts of these plant samples were prepared as aqueous, methanolic, ethanolic and aqueous ethanolic (25–75% v/v) extracts. The DPPH radical scavenging activity was determined using the method reported by Roy et al.^29^. DPPH was dissolved in methanol at a concentration of 50 μM. The DPPH solution (2.0 mL) was mixed with 2.0 mL of various concentrations (10, 20, 40, and 80 μg mL^−1^) of *S. chirata* extracts and immediately absorbance was measured at 517 nm using a spectrophotometer (UV2550, Shimadzu, Japan). Samples were incubated in a dark room for 16 min at 27 °C. After incubation, decrease in absorbance was again measured for all samples. Ascorbic acid was employed as a positive reference.

The scavenging activity was calculated using Eq. (1):

1 $$\mathrm{\% inhibition }= \frac{1-Ae}{Ac}\times 100$$

where Ae and Ac are absorbance of extract and control, respectively.

All experiments were performed using three replicates.

### Anti-diabetic assay (in-vitro)

In-vitro anti-diabetic activity of leaf parts of *S*. *chirata* samples harvested at bud- and flower-stages of plant was evaluated. Extracts of all plant samples were prepared by using different solvents viz. aqueous, methanolic, ethanolic and aqueous ethanolic (25–75% v/v). In-vitro anti-diabetic activity was assayed by means of method Roy et al.^29^. In this, inhibitory activity of α‑amylase enzyme was determined to check in vitro anti‑diabetic assay which involves the breakdown of starch into glucose. 1.0 mL of each *S. chirata* extracts (100 μg mL^−1^ of aqueous, methanolic, ethanolic and 25–75% aqueous ethanolic) were checked individually and into each test tube 1.0 mL of α‑amylase enzyme (Sigma, St. Louis, USA) was added and incubated at 37 °C for 10 min. After pre-incubation, 1.0 mL of 1% starch solution was added into each test tube. The reaction mixtures were again incubated at 37 °C for 15 min. Then the reaction was stopped with 2.0 mL of 3,5‑dinitrosalicylic acid (Sigma, St. Louis, USA) color reagent. The test tubes were then incubated in a boiling water bath for 5 min and then allow it to cool at room temperature, the absorbance was measured at 546 nm using a spectrophotometer. The control (buffer in place of sample extract) represents the 100% of enzyme activity.

The % age inhibition of α-amylase enzyme activity was calculated by Eq. (2):

2 $${\%} \text{ age\;inhibition\;of }\alpha\mathrm{-amylase }=\mathrm{ Enzyme\;activity\;of\;control}-\frac{\mathrm{Enzyme\;activity\;of\;extract}}{\mathrm{Enzyme}\;\mathrm{activity\;of\;control}}\times 100$$

All experiments were performed in triplicates and data were the mean ± SD. Statistical differences among groups were analyzed by one-way analysis of variance (ANOVA) followed by Duncan’s multiple range test (DMRT) by using SPSS 16. Groups were considered statistically significant at the significance level of *p* < 0.05.

### Ethics approval

The plant material for this study was collected from a nursery commercially which comply with relevant institutional, national, and international guidelines and legislation.

## Results

**Figure 1:**
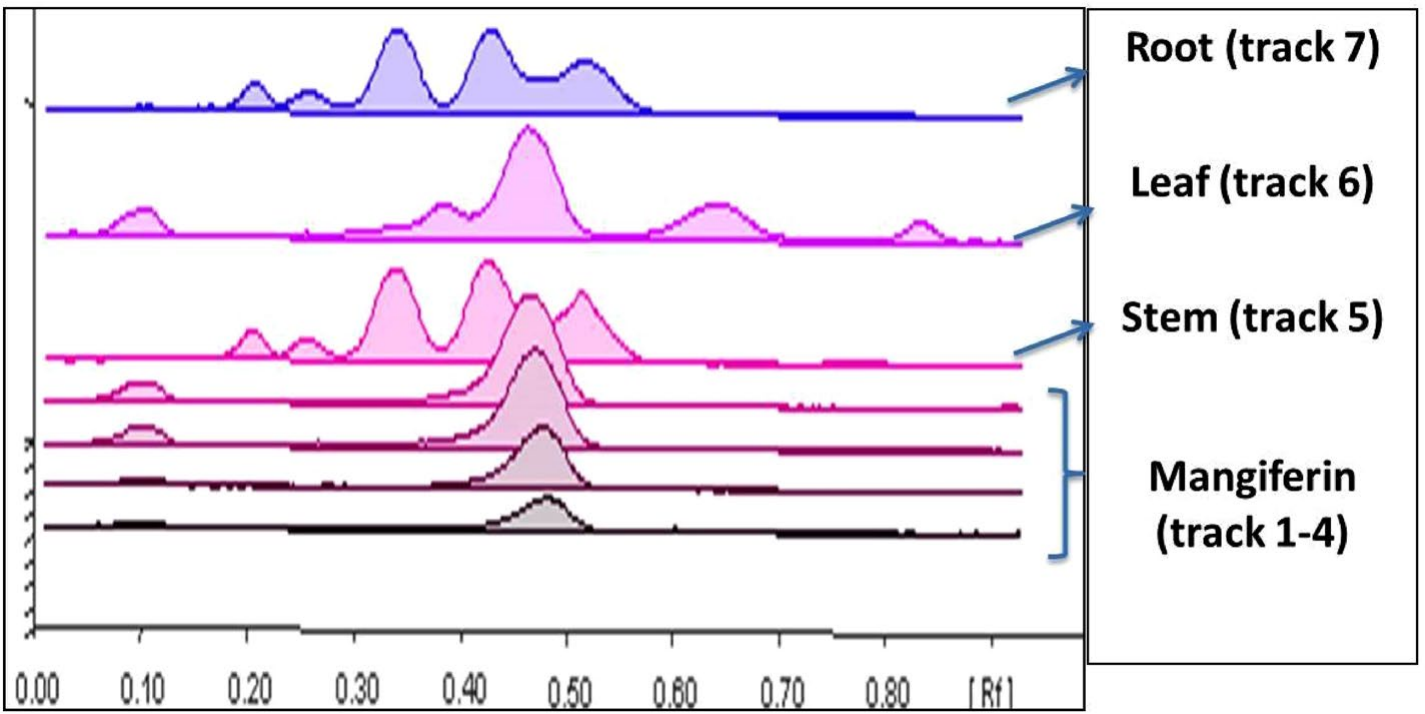
HPTLC profiles (overlay) of different plant parts of *Swertia chirata* (mobile phase: ethyl acetate-formic acid-glacial acetic acid–water: 100-11-11-26), Track 1–4 (standard mangiferin: 200 ng, 400 ng, 600 ng and 800 ng respectively, Rf-0.47), Track 5, 6 and 7 are stem, leaf and root, respectively.

In this study, the effects of plant parts, growth stage, drying method, storage periods, solvent type and the extraction method on the mangiferin content were investigated. Quantitative estimation of mangiferin in different extracts generated from different batches of *Swertia chirata* were identified by HPTLC fingerprinting. Although the HPTLC fingerprinting showed that mangiferin was present only in leaves part of the plant, it was decided to consider only the leaf part while excluding the stem and root parts (Fig. 1). In addition, HPLC–PDA analysis was used to confirm the HPTLC results.

**Figure 2:**
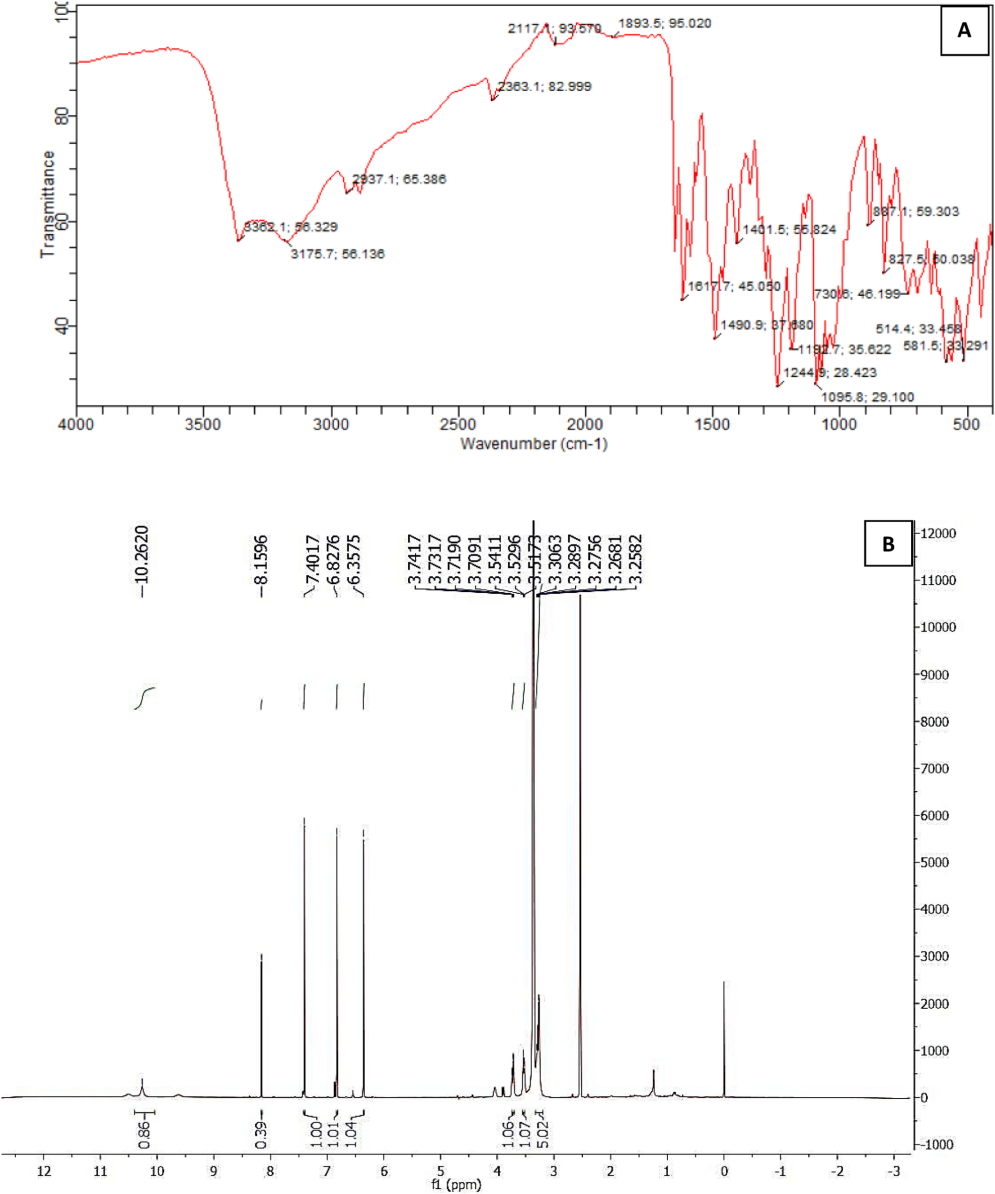
Characterization of isolated mangiferin fraction (F1) through IR (**A**) and ^13^C NMR (**B**).

**Figure 3:**
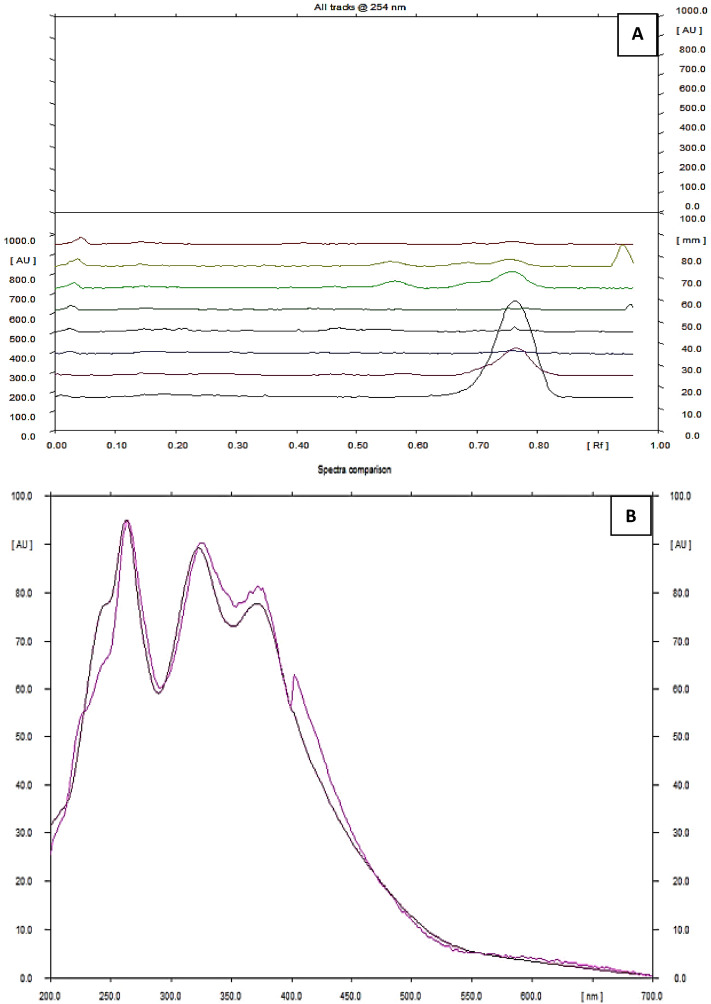
Comparison of reference compound (Track 1) with F1 (Track 2) by using Densitometric-HPTLC (**A**) and Overlay spectra (**B**) of reference compound with F1 at λ = 254.

In addition, flash chromatography was used to isolate the mangiferin compound from *Swertia chirata* leaf extract and out of seven fractions; mangiferin as the major peak was isolated as first fraction (FA) after residue at wavelength 254 nm. Fraction A containing mangiferin was confirmed and characterized for purity through IR and ^13^C NMR spectra (Fig. 2) and comparison with reference compound by using HPTLC (High performance thin-layer chromatography) and UV (Fig. 3).

Analytical characteristics of method validation parameters for mangiferin compound are presented in Table 3.

**Table 3:** Analytical characteristics of the method validation for mangiferin.

S. no.	Parameters	Mangiferin
1	Linearity range (ngspot^−1^; n = 12^a^	200–800
2	Correlation coefficient (r^2^)	0.987
3	Regression equation	Y = − 608.4 + 9.033X
4	Calculated SD value (CATS software)	6.72
5	^b^Limit of detection (LOD) (ng) [3 × SD/S]	25
6	^b^Limit of quantitation (LOQ) (ng) [10 × SD/S]	75
7	R_f_	0.47
8	λ_max (nm)_	258
**Precision and accuracy**		
7	Intra-day RSD (%), n = 5	0.437
8	Inter-day RSD (%),n = 5 (day-1/day-2/day-3)	0.437/0.495/0.512
**Recovery**		
9	Amount of mangiferin in leaf samples (μg mg^−1^) containing highest mangiferin	48.6
10	Amount of mangiferin added in leaf sample (μg mg^−1^)	20/40/60
11	Amount of mangiferin found (μg)	68.36 ± 0.251/88.4 ± 0.36/108.26 ± 0.35
12	Recovery (%) (mean)	99.99/99.77/99.68 (99.81)
13	RSD (%)	0.36/0.407/0.323

^a^Four concentration levels in triplicate; ^b^SD is the standard deviation of the blank response and S is the slope of the calibration plot.

### Influence of pre harvest factor (parts, growth stage) and post-harvest (drying methods and storage periods) factors on mangiferin content

Considering the optimal conditions for both pre-harvest and post-harvest factors on mangiferin yield; only the growth stages, drying methods and storage periods were used to optimize the mangiferin yield. The 3-way ANOVA tables were generated for mangiferin yield from *S. chirata* leaves and the significance of individual and interaction factors were examined. The significance of all the factors was observed statistically according to their p values. To visualize the impact of each factor-main and interaction plots were constructed.

The ANOVA analysis of the full factorial design data confirmed the expected significant effects of growth stages, storage periods and drying conditions on mangiferin yield. The ANOVA analysis further revealed that interaction of growth stage × storage periods was significant while other interactions viz. drying methods × growth stage and drying methods × growth stage × storage conditions were not significant. Table 1 shows the content of mangiferin in different samples of *S. chirata* at different growth stages, drying methods and storage periods. The Duncan’s multiple range test for mangiferin yield mean values and standard deviation (Table 1) showed that mangiferin yield at bud stage was significantly higher than the other growth stages of *S. chirata* plant.

**Figure 4:**
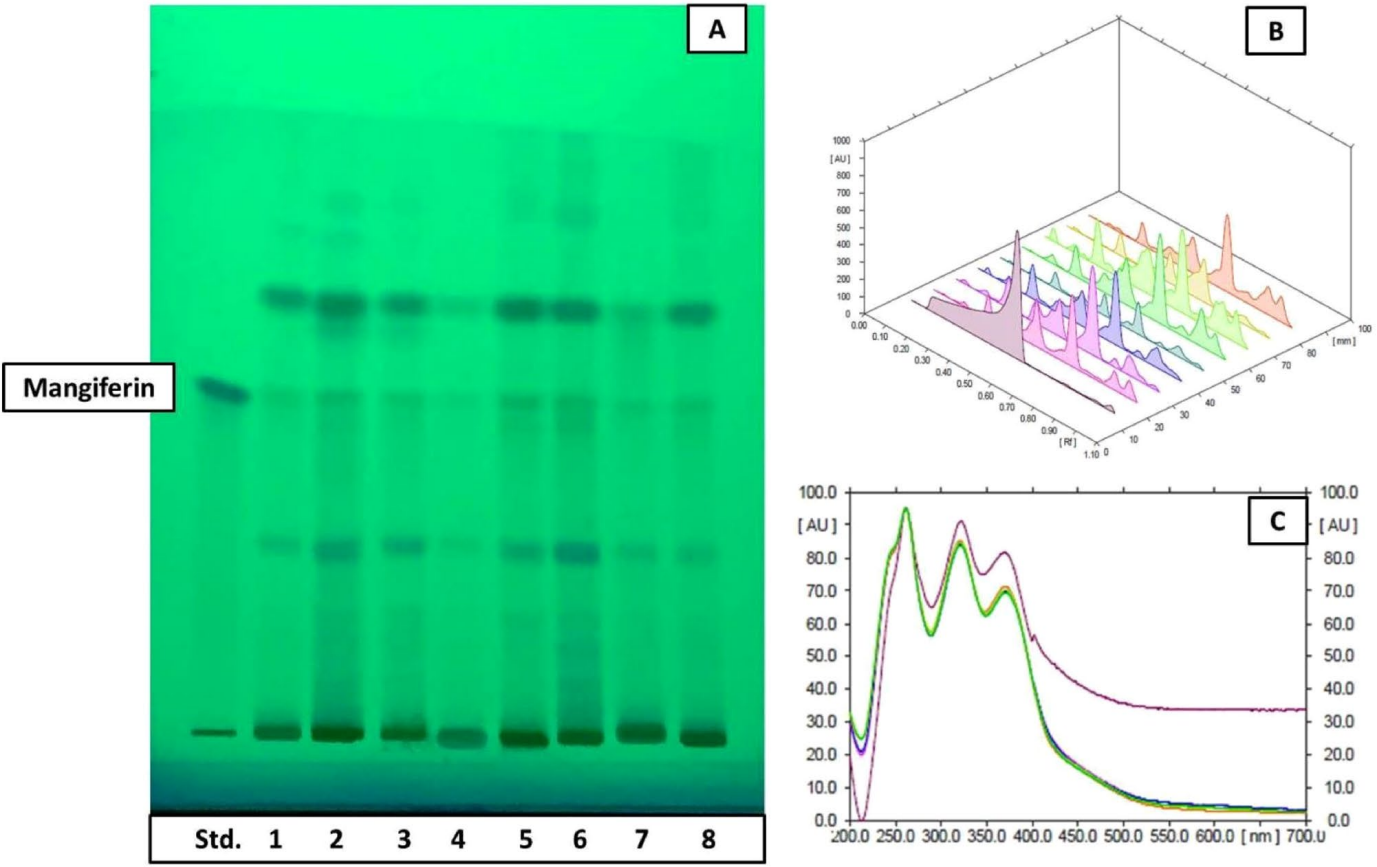
HPTLC fingerprinting (**A**) under UV_254_ light where tracks 1–3 represent sample prepared with HRE, UAE and MAE methods (using 50% EtOH); whereas 4–8 tracks represent sample harvested at different stages (Juvenile, Vegetative, Bud stage, Flower stage and Fruit stage, respectively) matched with standard compound-mangiferin (1st spot); (**B**,**C**) demonstrate 3-D densitogram and overlay spectra of *Swertia chirata* test samples compared with standard compound (mangiferin).

**Figure 5:**
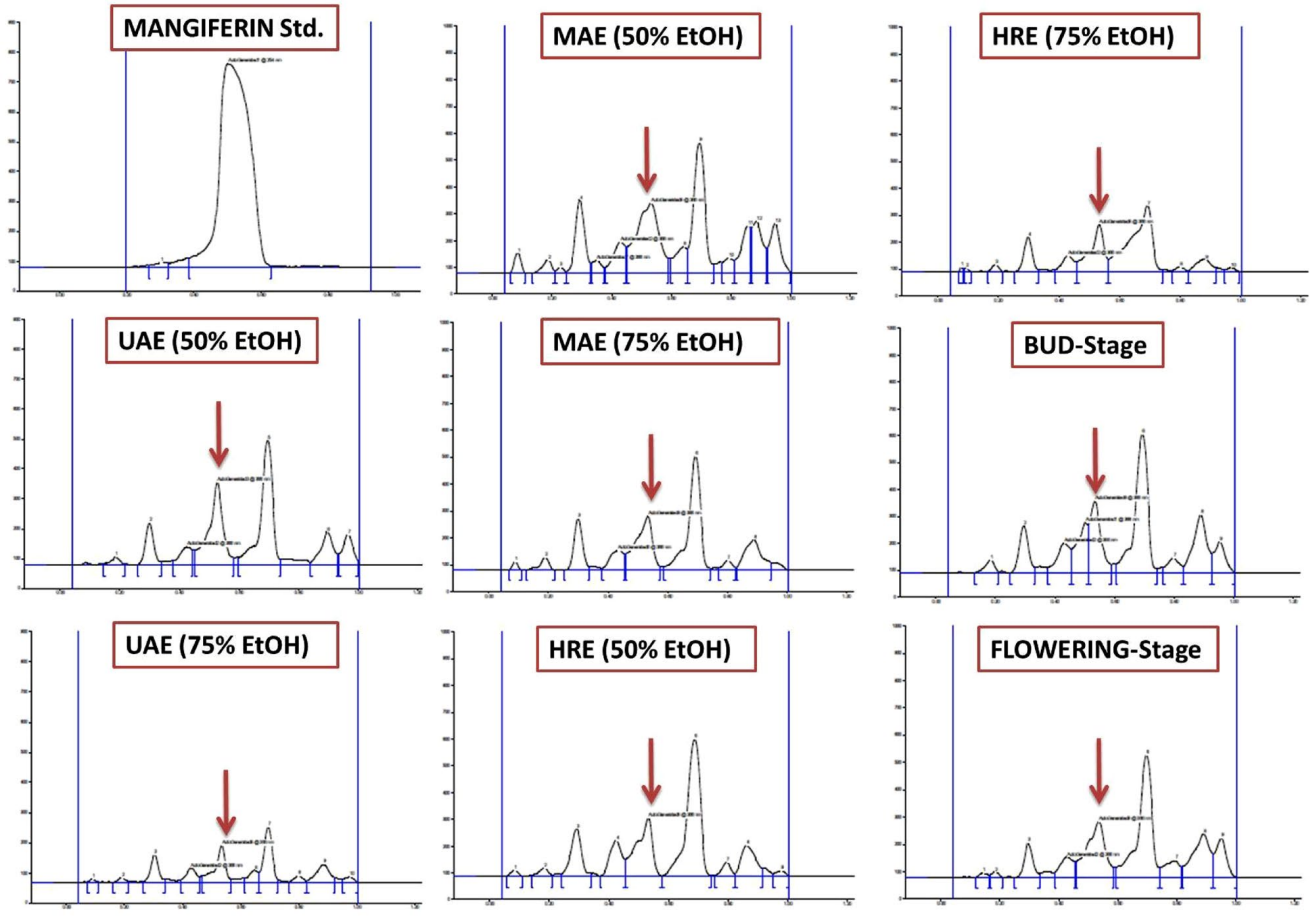
Densitometric-HPTLC chromatograms of *S. chirata* demonstrating effect of extraction methods viz. ultrasound-assisted extraction—UAE, microwave-assisted extraction—MAE, heat-reflux extraction—HRE and stage of plant on mangiferin yield.

**Figure 6:**
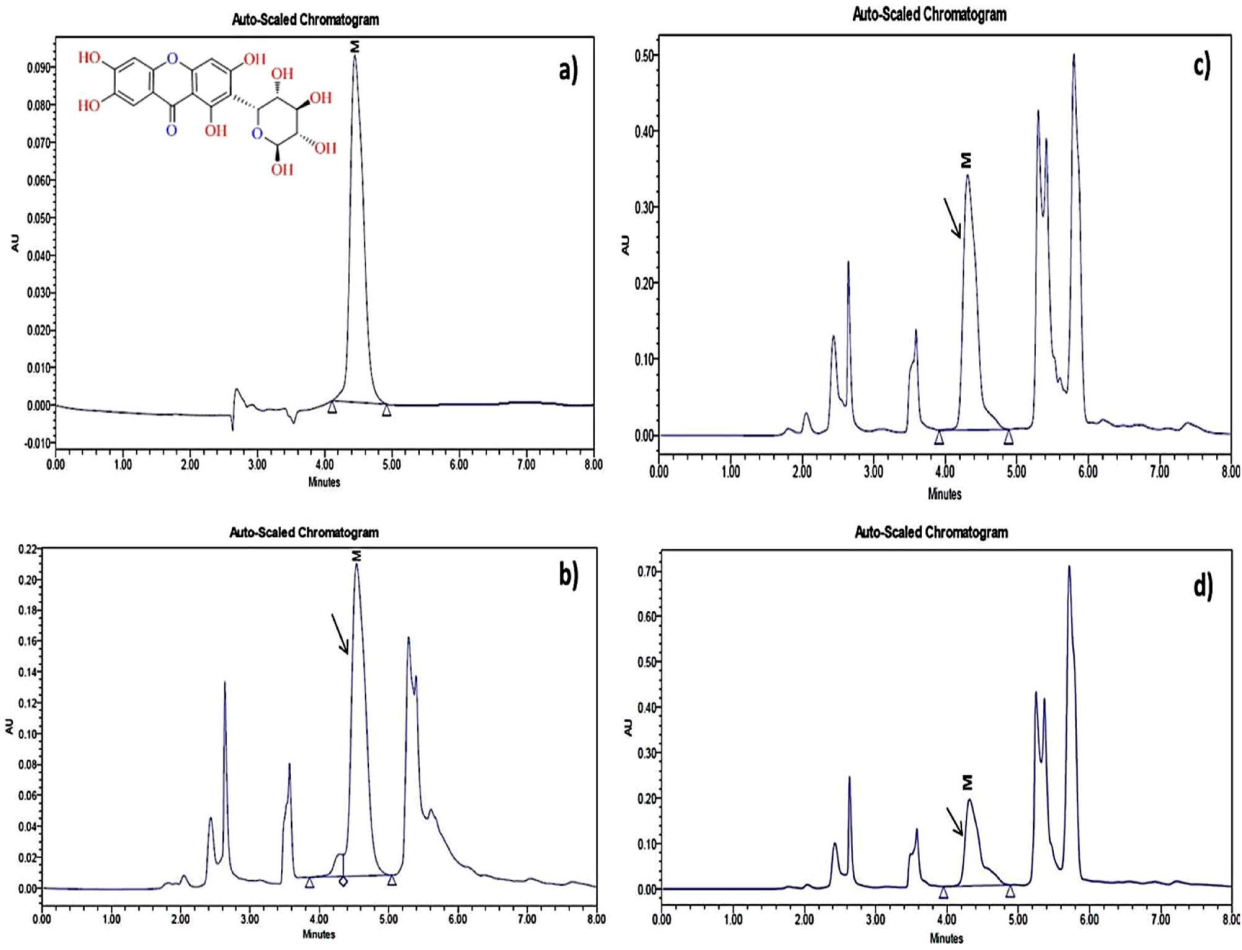
HPLC chromatogram of a mangiferin: standard compound (**a**) whereas (**b**), (**c**,**d**) represents HPLC chromatograms obtained from *S. chirata* samples prepared with ultrasound-assisted extraction (UAE), microwave-assisted extraction (MAE) and heat reflux extraction (HRE), respectively by using 50% EtOH solvent composition.

**Figure 7:**
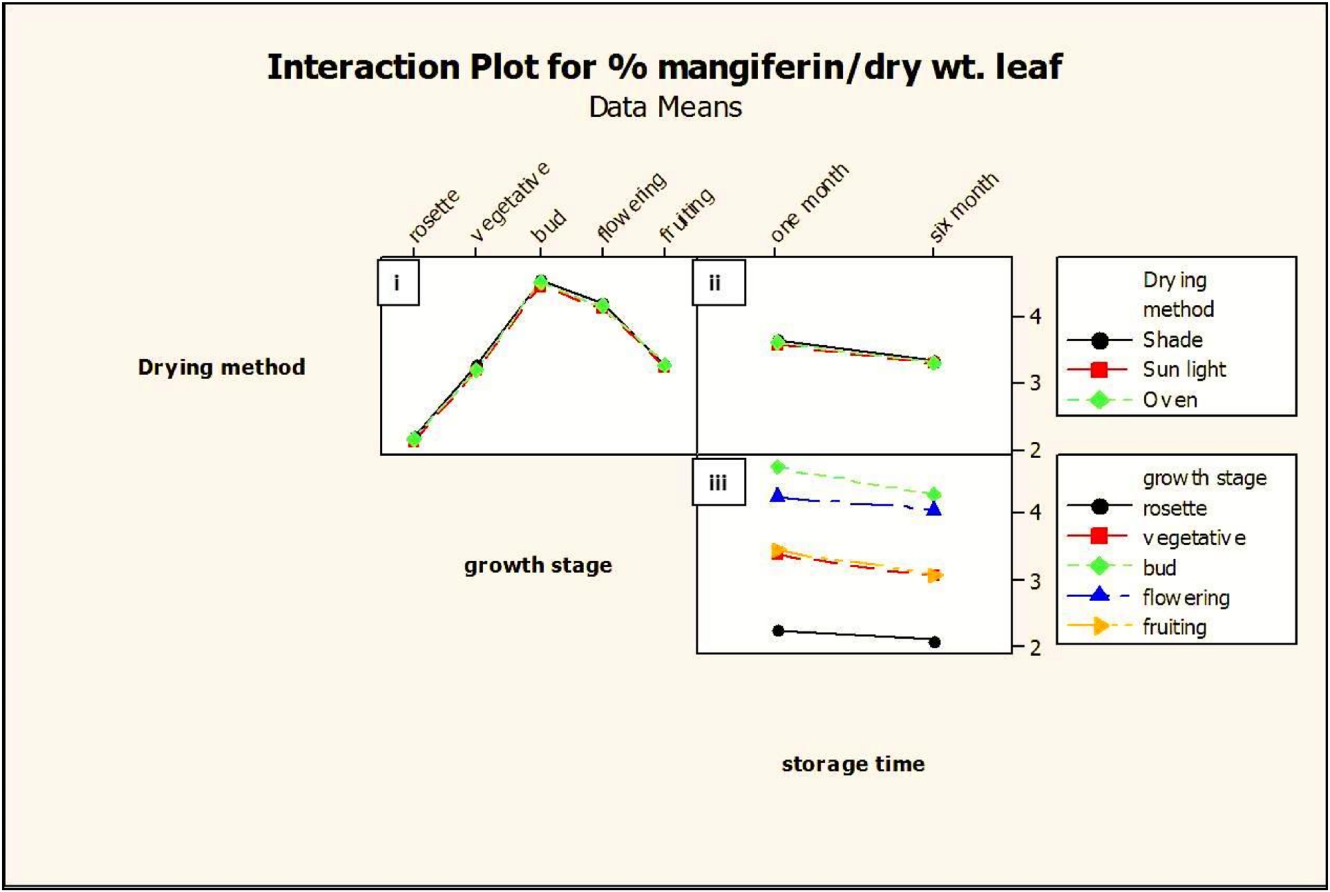
Interaction plot of growth stage, drying method and storage period for mangiferin yield from *Swertia chirata* leaves. Dot is the mean value of mangiferin content.

**Figure 8:**
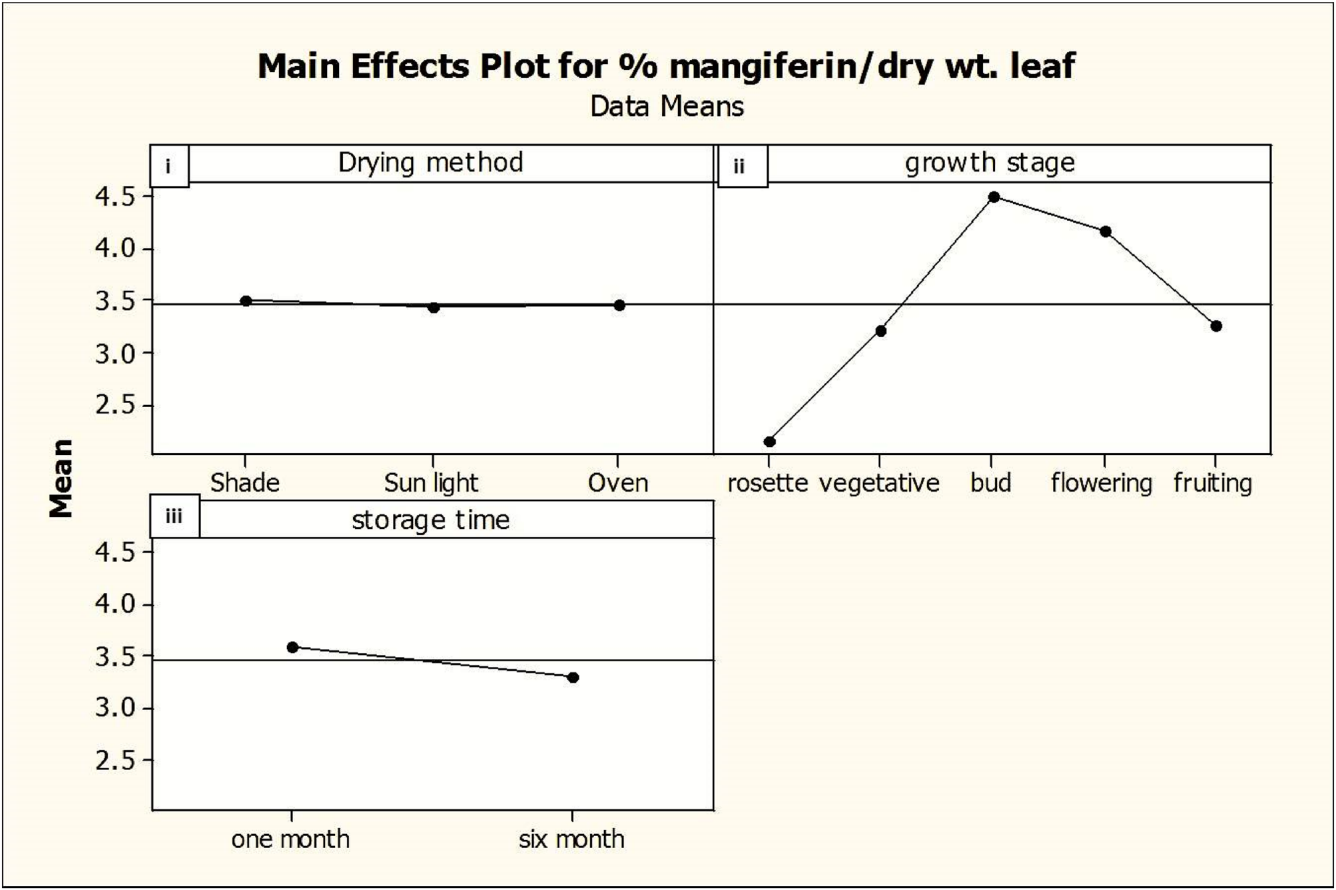
Main plots of growth stage, drying method and storage periods for mangiferin yield from *Swertia chirata* leaves. Dot is the mean value of mangiferin content.

HPTLC fingerprints obtained under UV_254_ light, 3-D Densitogram, overlay spectra and Densitometric-HPTLC chromatograms of *S. chirata* test samples compared with standard compound (mangiferin) are shown in Figs. 4 and 5. Figure 6 presents HPLC chromatogram of mangiferin: standard compound with HPLC chromatograms obtained from *S. chirata* samples prepared with ultrasound-assisted extraction (UAE), microwave-assisted extraction (MAE) and heat reflux extraction (HRE), respectively by using 50% EtOH solution. Interaction and main plots of growth stage, drying methods and storage periods for mangiferin yield from *S. chirata* leaves are presented in Figs. 7 and 8.

#### Effect of pre-harvest stage (growth stage and season) on mangiferin content

Mangiferin content was varied from 2.32 to 4.73% in different growth stages of the plant. Lowest mangiferin concentration (2.293%) was detected at the rosette stage (juvenile growth phase) of the plant whereas maximum mangiferin yield (4.73%) was observed in the bud stage of *Swertia* plant. As the flowering period occurred in *Swertia* during the September month, mangiferin yield gradually started declining to 4.31% and eventually its concentration lowers to 3.45% in the fruiting stage of herbs during the end of November (Table 1, Figs. 7, 8).

#### Effect of post-harvest (drying methods) on mangiferin content

Mangiferin from the leaves samples of *Swertia chirata* kept for drying under different conditions were determined (Table 1). Comparison of the results showed that different drying methods had a significant effect on mangiferin content (Figs. 7, 8). The relative proportions of mangiferin of shade-dried leaves were higher than the other two drying methods. It could be concluded that drying of leaves of *S. chirata* in the shade drying was more suitable than oven and sun drying and is recommended for obtaining higher yield of mangiferin. The effect of the sun drying was more pronounced with a large decrease (4.663%) of mangiferin as compared to oven drying (4.717%) of mangiferin in 1-month old stored leaves harvested at bud-stage (4.73%).

#### Effect of post-harvest (storage periods) on mangiferin content

An attempt has been made to check the degradation of mangiferin compound under different storage periods after harvesting. Powdered samples of *S. chirata* leaves were stored in polypropylene containers and kept in dark at room temperature. HPTLC reports revealed that there were no significant variations of mangiferin content in plant samples immediately after drying and storing up to 1 month in our studies but subsequently there was declined in the mangiferin content in dried leaves. The comparison of the two-storage time revealed that total mangiferin contents of 1-month old plant sample are higher than those of 6-month-old samples. The results displayed that mangiferin content in the 1-month-old leaves showed 4.73% mangiferin as compared to 4.34% in 6 months old *S. chirata* leaves powder (Figs. 7, 8) showing that there was significant variation of mangiferin content in all the plant samples stored under controlled conditions for 1 month and 6 months. These results are according with those reported by Manika et al*.*^18^ at the same conditions.

#### Preliminary experiment for screening of solvents to better yield of mangiferin

Mangiferin, as C-glucoside polyphenol compound, is soluble in medium-polar solvents i.e. ethyl acetate, dichloromethane and polar solvents i.e. ethanol, methanol, water. Preliminary extraction conditions for screening the best solvents were optimized by using different polar solvents viz. chloroform, ethyl acetate, methanol, ethanol, 50% methanol, 50% ethanol and distilled water. Extraction of mangiferin from *Swertia chirata* leaves (1 g) by refluxing with different solvent composition such as chloroform (CHCl_3_), ethyl acetate (EtOAc), methanol (MeOH), MeOH:CHCl_3_ (5:5), MeOH:EtOAc (5:5), ethanol (EtOH), 50% EtOH and H_2_O for 1 h at 75 °C yielded 0.6%, 0.83%, 2.11%, 1.21%, 1.56%, 1.46%, 4.14%, 2.75% mangiferin in *S. chirata* leaves (Table 2). Ethanol is recommended as non-toxic solvent and also yielded highest content of mangiferin in *S. chirata* leaves. Mangiferin content in *S. chirata* at room temperature by methanol solvent was found to be 2.16%. Hence, ethanol instead of methanol could be used as an effective solvent for extracting the significant phytochemical marker- ‘Mangiferin’ for obtaining the highest content from *S. chirata* leaves.

Effect of different compositions of aqueous ethanol on mangiferin yield from *S. chirata* leaves are presented with Densitometric-HPTLC chromatograms (Fig. 5).

### Optimization of extraction conditions by full factorial design

Selection of effective extraction method is a key concern for the recovery and isolation of desired bio-active compounds from a mixture of crude matrix. The present work attempts to compare the heat reflux (HRE), microwave assisted (MAE) and ultrasound assisted extraction (UAE) techniques to determine the efficient method to extract mangiferin from *S. chirata* leaves. The extraction efficacy of the different techniques has been varied between 1.46 and 4.86% (Table 2).

**Figure 9:**
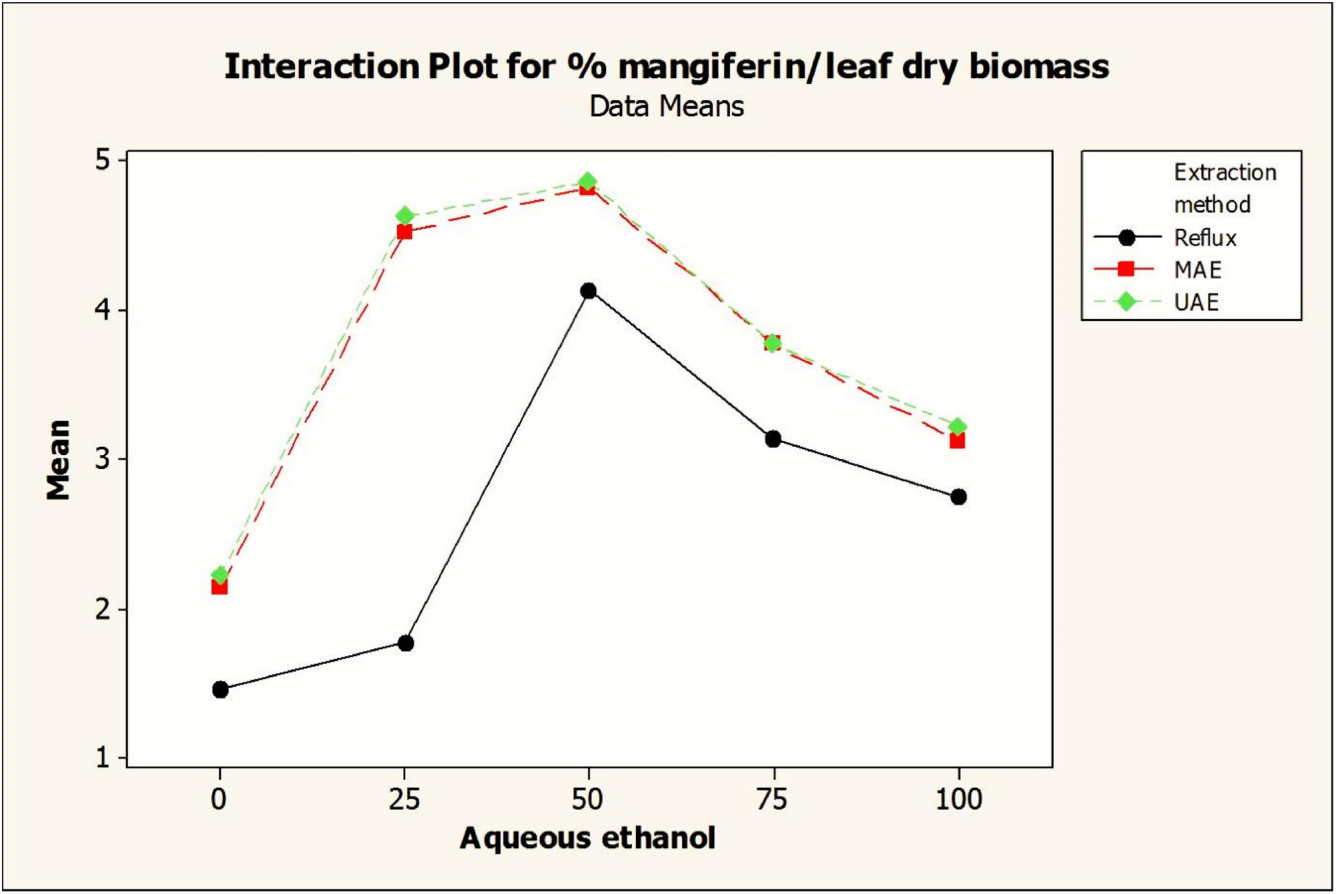
Interaction plot of extraction methods and aqueous ethanol composition for mangiferin yield from *Swertia chirata* leaves. Dot is the mean value of mangiferin content.

**Figure 10:**
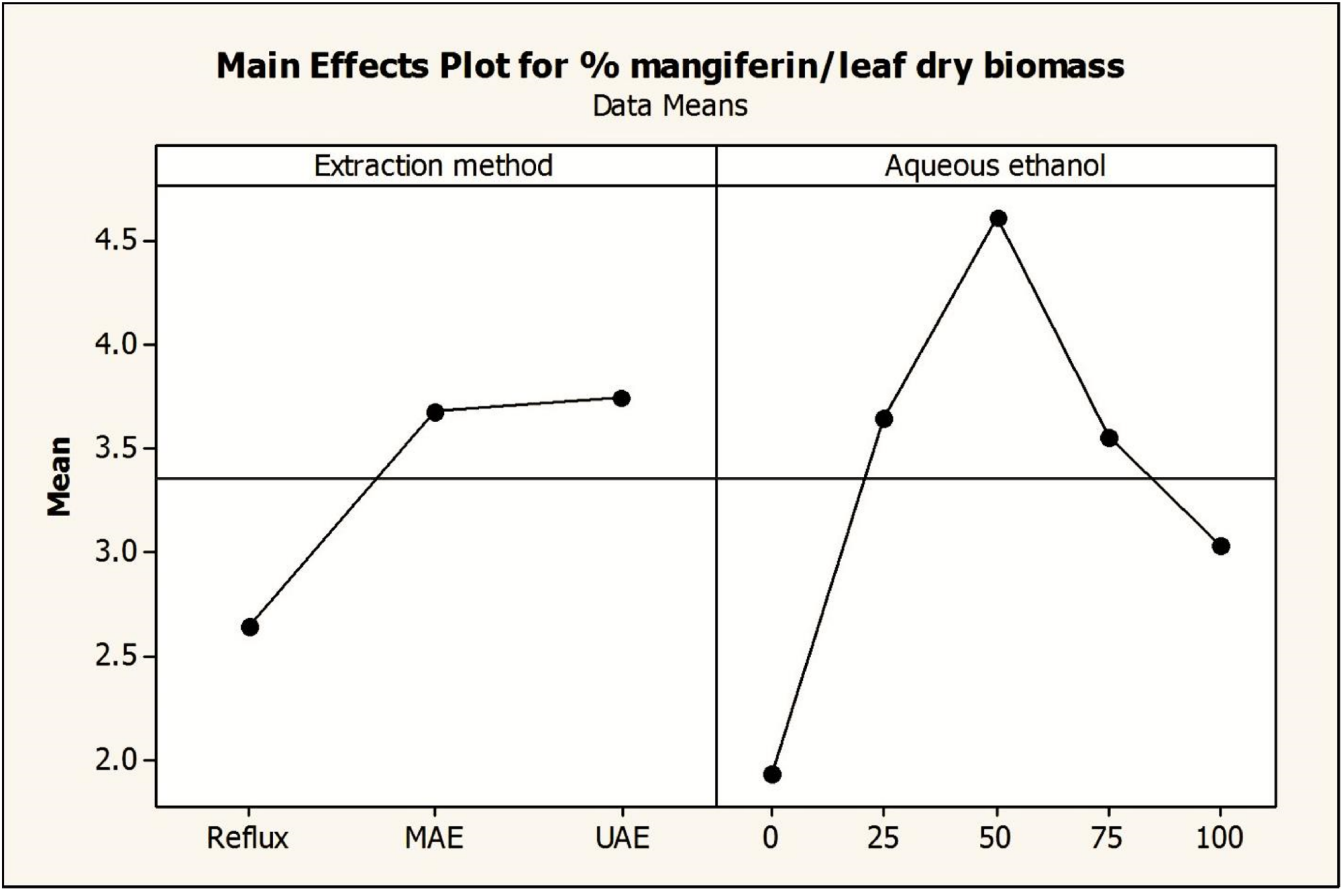
Main plots of extraction methods and aqueous ethanol composition for mangiferin yield from *Swertia chirata* leaves. Dot is the mean value of mangiferin content.

Two-way ANOVA tables were generated for the determination of significance of individual and interactive factors for mangiferin yield from *S. chirata* leaves. On the basis of *p* values (≤ 0.05) the significance of all the factors were judged statistically (Table 4). Main effect and interaction plots were constructed to study the most influential factors affecting the mangiferin yield (Figs. 9, 10). The ANOVA analysis of the full factorial design data confirmed the expected significant effects of extraction type (HRE, MAE, UAE) and also revealed the significant impact of aqueous ethanol solvent composition on mangiferin yield from *S. chirata* (Table 2). The Duncan’s multiple range test for mangiferin yield mean values showed for *S. chirata* leaf extracted with UAE and MAE was better than HRE. Heat refluxing with different solvent compositions such as absolute ethanol, 25% aqueous ethanol, 50% aqueous ethanol, 75% aqueous ethanol and aqueous for 1 h at 75 °C yielded 1.46%, 1.77%, 4.14%, 3.137% and 2.75%, respectively. Subsequently, microwave assisted extraction (MAE) and ultra-sonicator assisted extraction (UAE) methods were applied to extract mangiferin from *S. chirata* leaf using same solvent compositions as that of reflux method and these methods were found to be better than the reflux extraction. In the experimentation, UAE has shown highest mangiferin content 4.86% as compared to MAE (4.82%) and HRE (4.14%) methods.

**Table 4:** Analysis of Variance for solvents and extraction techniques on mangiferin yield from *Swertia chirata* leaves.

S. no.	Extraction factors	DF	Seq SS	Adj SS	Adj MS	F	P
1	Extraction methods	2	11.2078	11.2078	5.6039	9136.75	0.000
2	Aqueous ethanol	4	34.1103	34.1103	8.5276	13,903.67	0.000
3	Extraction methods × aqueous ethanol	8	7.7574	7.7574	0.9697	1580.98	0.000
4	Error	30	0.0184	0.0184	0.0006		
5	Total	44	53.0939				
	S = 0.0247656 R-Sq = 99.97% R-Sq(adj) = 99.95%

HPTLC fingerprints obtained under UV_254_ light, 3-D Densitogram, overlay spectra, Densitometric-HPTLC chromatograms and HPLC chromatograms of *S. chirata* test samples compared with standard compound (mangiferin) are shown in Figs. 4, 5 and 6. Interaction and main plots depicting the effects of different extraction methods (HRE, MAE and UAE) with aqueous ethanol compositions (25–100%) on mangiferin yield from *Swertia chirata* leaves are presented in Figs. 9 and 10.

#### Effect of HRE and aqueous ethanol on mangiferin yield

Results of mangiferin content using HRE techniques are shown in Table 2. The results showed that mangiferin yield varies with the aqueous ethanol composition by keeping the extraction time 1 h. An increasing trend in yield of mangiferin with respect to increase in polarity of solvent was observed from 100% ethanol to 50% aqueous ethanol after that there was a decrease in the yield of mangiferin at 75% aqueous ethanol and 100% aqueous (Figs. 9, 10). The high temperature facilitates the dissolution and displacement of saturation equilibrium constant and increases efficiency of extractable compounds.

#### Effect of MAE and aqueous ethanol on mangiferin yield

MAE resulted in higher yield of mangiferin as compared to HRE method, ranging from 2.147 ± 0.09 to 4.820 ± 0.19 (Table 2). The pattern observed (highest at 50% aqueous ethanol) is similar to that observed in HRE. The results of the effect of aqueous ethanol on yield of mangiferin are presented in Figs. 9 and 10.

#### Effect of UAE and aqueous ethanol on mangiferin yield

Content yield vary from 2.22 ± 0.091 to 4.860 ± 0.19 (Table 2). Ultra sonicator exposure for 30 min showed best mangiferin yield at 50% aqueous ethanol. Effect of aqueous ethanol on yield of mangiferin is depicted in Figs. 9 and 10.

### Anti-oxidant and anti-diabetic activity

The antioxidant and in-vitro anti-diabetic activities were determined from different extracts of leaves of *S. chirata* harvested at bud- and flower-stage of plant. The results from both assays showed significant differences in the anti-oxidant and anti-diabetic activities among the different sample conditions and solvent type (Table 5), with aqueous ethanol (50%) extracts having greater DPPH and percentage inhibition of α‑amylase activity than the methanolic, ethanolic and aqueous extracts. The DPPH activity was in the range of 62.5–82.1% with the highest and lowest DPPH activity observed in the aqueous ethanol (50%) extracts of the leaves harvested at bud-stage and ethanol extracts of the leaves harvested at flowering stage, respectively. Different concentrations of DPPH (10–80 μg mL^−1^) were used to calculate the anti-oxidant activity of plant samples, and it was observed that percentage increases sharply with increased concentration of DPPH. At a concentration of 80 μg mL^−1^, the highest DPPH activity was observed in the aqueous ethanol (50%) extract of *S. chirata* leaves harvested at bud-stage (82%) of plant followed by flowering stage (78%).

**Table 5:** Antioxidant and anti-diabetic activity of *S. chirata* leaves harvested at different plant phases and extracted with different solvents.

Plant	Harvesting stage	Solvents	DPPH (%)	Percentage inhibition of α‑amylase
*S. chirata*	Bud-stage	Methanol Ethanol	67.3 ± 3.89^b,c^ 65.4 ± 2.21^b,c^	75.2 ± 3.42^a,b^ 74.5 ± 2.12^a,b^
		Aqueous ethanol (50%)	82.1 ± 4.01^a^	77.6 ± 2.23^a^
		Aqueous	70.2 ± 2.23^b^	72.1 ± 4.54^a,b^
	Flower-stage	Methanol Ethanol	64.1 ± 3.54^b,c^ 62.5 ± 2.06^c^	73.3 ± 4.11^a,b^ 72.7 ± 2.29^a,b^
		Aqueous ethanol (50%)	78.4 ± 4.25^a^	74.2 ± 3.04^a,b^
		Aqueous	68.2 ± 3.24^b,c^	69.5 ± 3.25^b^

Moreover, percentage inhibition activity of α‑amylase was observed in the range of 69.5–77.6% with the maximum and least in-vitro activity observed in the aqueous ethanol (50%) extracts of the leaves harvested at bud stage and ethanol extracts of the leaves harvested at flowering stage, respectively (Table 5). Among all the plant samples, highest percentage inhibition of α‑amylase was observed in the aqueous ethanol (50%) extract of *S. chirata* leaves harvested at bud-stage (77%) followed by flowering stage (74%) plant.

All analyses are the mean of three replicates measurements ± standard deviation. The results were analyzed by one-way ANOVA followed by Duncan’s Multiple Range Test. Values with different alphabets (a–c) and (a–b) within the same column (DPPH %) and (% α-amylase activity) are significantly different at *p* < 0.05 respectively.

## Discussion

The effects of plant parts, growth stage, drying method, storage periods, solvent type and the extraction method on the mangiferin content were investigated. Earlier reports revealed the significant variation of mangiferin yield in *Swertia* spp. leaves amongst rosette and fruiting stage of plant that were harvested between June and November months. Yang et al.^30^ reported the highest content of mangiferin in a bud-stage of *Swertia mussoti (*a potent Chinese *Swertia* species) herb but showed no variation between flowering and fruiting stage. It was already reported that content of bio-active compound varies with growth stage of plant, seasonal variation/and proper harvesting time period^31,32^. In other medicinal plants, some researchers also observed the gradual seasonal variation in various bio-active compounds^18,31,33–35^. Many previous researchers reported the importance of drying methods and their circumstances on the amount and quality of the phytochemicals. All these studies show the superiority of shade drying over the oven drying, sun drying and other drying methods of the plant samples^36^. As far as selection of solvent were concern ethanol is recommended as non-toxic solvent and also yielded highest content of mangiferin in *Swertia chirata* leaves (Figs. 3, 4, 5, 8, 9, 10). Hence, ethanol instead of methanol could be used as an effective solvent to extract the significant phytochemical marker-‘Mangiferin’ to obtain the highest content of these compounds from *S. chirata* leaves^19^. Mangiferin content in *Swertia chirata* at room temperature by methanol solvent was found to be 2.16% with the method described by Pandey et al.^28^. Mangiferin, a C-glucoside polyphenol compound is soluble in medium-polar solvents i.e. ethyl acetate, dichloromethane and polar solvents i.e. ethanol, methanol, water.

The high temperature facilitates the dissolution and displacement of saturation equilibrium constant and increases efficiency of extractable compounds. Ruiz-Montanez et al.^37^ extracted mangiferin by using ethanol–water (8:2 v/v) by soxhlet from mango peel. When comparing the time needed to achieve the complete extraction of mangiferin, HRE takes longer time (60 min) followed by UAE (30 min) and MAE (2 min) at 50% aqueous ethanol. Similar mangiferin yield was obtained in a plant sample by using method of Pandey et al.^28^ at room temperature by soaking leaf powder in methanol for 24 h. This is because an increase in temperature in HRE facilitates the dissolution of mangiferin and penetration of aqueous ethanol solvent in plant matrix more than methanol solvent in short duration^27^. Microwave assisted extraction (MAE) uses microwave radiations for better extraction by directly affecting the molecules by dipole polarization and thus rapidly heats the solvent^22^. UAE uses ultrasound waves to rupture the cell wall due to the micro-cavities in the plant material and thus resulting in the efficient extraction of bio-active compounds^23^. When comparing the time needed to achieve the complete extraction of mangiferin, HRE takes longer time (60 min) followed by UAE (30 min) and MAE (2 min) at 50% aqueous ethanol.

In consequence UAE and MAE methods resulted in the highest yield of bio-active compounds and also supposed to be a cost effective, rapid and green extraction technology. In future, UAE and MAE methods can be efficiently used to get the maximum mangiferin content from *S. chirata* leaves.

The study found that binary solvent mixture (aqueous ethanol) resulted in better yield of polyphenols^38^. Ethanol breaks the bond between solute and plant materials, reduces surface tension of the medium and increases the mass transfer of bioactive compounds into the solvent; on the other hand water causes cell swelling to increase the surface area^39^. In the present study, aqueous ethanol was also found to give maximum anti-oxidant and anti-diabetic activity, which is higher than water, absolute ethanol and methanol. Mangiferin, as xanthonoid is the major phytochemical compound of *S. chirata*, which is well known to exhibit anti-oxidant and anti-diabetic activity. As the present study concluded that only aqueous ethanol produced the highest yield of mangiferin in *S. chirata* plant samples, consequently it was coherent that the plant leaves extracted with the same solvent had the highest anti-oxidant and anti-diabetic activities.

## Conclusions

The experimental design approach using full factorial design was successfully applied in the optimization of mangiferin yield from *Swertia chirata* leaves by using HPTLC and HPLC. Firstly, full factorial design for optimization of pre-harvest and post-harvest factors was evaluated and improved mangiferin yield (4.73%) was only found in *S. chirata* test samples harvested at bud-stage and shade dried for 1 month. Furthermore, optimum mangiferin yield (4.86%) was obtained by choosing an extraction method, UAE and 50% aqueous ethanol solvent solution. In this study, appropriate growth stage at budding, leaf part, shade drying and storage period for 1 month, UAE and 50% aqueous ethanol were found to be significant factors to achieve the highest yield of mangiferin from *Swertia chirata*.

## Data Availability

The datasets used and/or analyzed during the current study available from the corresponding author on reasonable request.

## Abbreviations

HPTLC: High performance thin layer chromatography
HPLC: High performance liquid chromatography
